# How Well Do Older Adult Fitness Technologies Match User Needs and Preferences? Scoping Review of 2014-2024 Literature

**DOI:** 10.2196/75667

**Published:** 2025-09-24

**Authors:** Christopher Tacca, Arturo Vazquez Galvez, Isobel Margaret Thompson, Alexander Dawid Bincalar, Christoph Tremmel, Richard Gomer, Martin Warner, Chris Freeman, m.c schraefel

**Affiliations:** 1 School of Electronics and Computer Science Faculty of Engineering and Physical Sciences University of Southampton Southampton United Kingdom; 2 School of Health Sciences University of Southampton Southampton United Kingdom

**Keywords:** elder physical fitness, fitness technologies, digital health, physical activity promotion, older adults, mobile phone

## Abstract

**Background:**

The population is aging, and research on maintaining older adult independent living is growing in interest. Digital technologies have been developed to support older adults’ independent living through fitness. However, reviews of current fitness technologies for older adults indicate that the success is considerably limited.

**Objective:**

This scoping review investigates older adult fitness by comparing current interventions to known needs and preferences of older adults from older adult–specific technology acceptance research, barriers and enablers to physical activity, and qualitative research on fitness technologies. The review questions are (1) How well do current older adult fitness technologies align with known preferences? (2) How well do current research methodologies evaluate the known needs and preferences?

**Methods:**

Research papers from the last 10 years were searched in the ACM Digital Library, IEEE Xplore, Medline, and PsycINFO databases using keywords related to older adults, technology, and exercise. Papers were only included if they specifically evaluated fitness technologies, focused on older adults, and mentioned a specific technology used in the intervention. To evaluate the fitness interventions, an assessment tool, the Older Adult Fitness Technology Translation Assessment tool, was synthesized through literature on technology acceptance, barriers and enablers to physical activity, and qualitative research on fitness technologies. Interventions were scored by 5 reviewers using a dual-review approach.

**Results:**

A total of 43 research papers were selected:16 from medical journals, 15 from engineering journals, 7 from human-computer interaction journals, 3 from public health, and 2 from combined computing and engineering journals. The Older Adult Fitness Technology Translation Assessment tool contained six assessment factors: (1) compatibility with lifestyle, (2) similarity with experience, (3) dignity and independence, (4) privacy concerns, (5) social support, and (6) emotion. The average scores of the 6 factors were 2.93 (SD 0.86) on compatibility with lifestyle, 3.10 (SD 0.74) on similarity to experience, 3.49 (SD 0.64) on dignity and independence, 3.17 (SD 0.86) on privacy concerns, 3.74 (SD 0.81) on short-term outcomes, 2.75 (SD 1.21) on long-term outcomes, 2.79 (SD 0.88) on social support, and 3.17 (SD 1.19) on emotion. No research paper scored a 3 or above on all 6 factors.

**Conclusions:**

The results show a lack of alignment between the known preferences of older adults and the design and assessment of current older adult fitness technologies. Areas for growth include (1) alignment between the needs of older adults and fitness technology intervention design, (2) translation of findings from older adult design work to designs in practice, and (3) explicit usage of older adult–specific factors in research. We hypothesize that the proposed Older Adult Fitness Technology Translation Assessment tool can help bridge the gap between technological capability and real-world applicability, ultimately fostering greater acceptance, respect, and long-term success.

## Introduction

### Background

The global population is aging [1,2]. Care needs for older adults, including maintaining and extending older adult independent living, have likewise grown as an area of research focus [1,2]. An approach toward building and sustaining quality of life is to develop digital technologies to support fitness practices such as mobility, strength building, and aerobic health, defined as fitness technologies. The rationale for this technology-supporting fitness approach notes that the benefit for older adult independence in improving overall fitness in general [3,4] and strength building in particular [5] is well-established; and that, likewise, the scalability and affordability of fitness technologies relative to in-person specialist care [6] would enable such fitness support to be more accessible to more older adults.

In the last 7 years, there have been no less than 8 related review papers around computational health interventions for older adults including: wearable devices for increasing physical activity [7], mHealth and eHealth solutions for increasing physical activity [8], smart technology interventions for increasing physical activity in comparison to face-to-face interventions [9], eHealth interventions for increasing balance and preventing falls [10], virtual reality (VR) based interventions for improving functional mobility in older adults [11], adherence of technology-based exercise interventions [12], and older adult experiences using wearable-based interventions [13,14].

Crucially, the conclusions of these reviews, summarized in Table 1 and discussed in the Related Work section, indicate that successes in this area seem to be relatively mixed. Findings from the review are characterized as very low to moderate evidence of effectiveness [8,9], high risk of bias [7], and low methodological quality [10-12].

**Table 1:** Summary of the outcomes of relevant reviews in the field.

Review authors	Subject	Outcomes
Cooper et al [7]	Effectiveness of wearable-based interventions for physical activity promotion	Accelerometers increased physical activity, but pedometers did not; high risk of bias
McGarrigle and Todd [8]	Effectiveness of eHealth and mHealth for physical activity promotion	Low to moderate evidence to increase physical activity
D’Amore et al [9]	Comparison of smart technology versus face-to-face interventions for physical activity promotion	May improve daily step counts, but very low evidence
Gaspar and Lapão [10]	Effectiveness of eHealth for increasing balance	Potential for balance improvements and fall risk reduction, but with no clear comparison
Corregidor-Sánchez et al [11]	Effectiveness of virtual reality for functional mobility	Virtual reality is effective compared to conventional treatment, but with low methodological quality
Valenzuela et al [12]	Adherence to technology-based exercise programs	High adherence through the first 12 weeks of intervention, but with methodological problems, including sample size, inclusion criteria, and follow-up times
Moore et al [13]	Older adults’ experiences with wearable-based interventions related to physical activity and fall prevention	Emergent themes of older adults’ use of wearables include motivation, user characteristics, integration into daily life, and device features
Vargemidis et al [14]	Older adults’ experiences with wearable physical activity interventions with a focus on the design and evaluation processes	Most interventions focus on supervising older adults, whereas supporting older adults’ involvement in the design process is scarce

Given both the increasing need for such work and the growing interest across disciplines, it is concerning that these reviews, summarizing work across a range of technologies from accelerometers to VR, do not evidence stronger successes and impact. The focus of this scoping review, therefore, has been to determine what the factors may be in common across this research that may be hindering better outcomes. To interrogate this problem, we took a dual approach to our review and its analysis:

First, we reviewed research from across disciplines that used technology to help older adults build strength and maintain their independence. As such, the main criteria for inclusion are that a novel technology-based intervention was intended to support or improve fitness for older adults. From this collection, we then considered a range of shared quantitative features such as: technologies used, types of evaluations, whether co-design is part of the process, and duration of evaluations. Each of the metrics is described in the Methods section below.

Second, we developed an assessment tool drawing from research that identifies the needs and preferences of older adults with respect to technology and physical fitness to assess current older adult fitness technologies’ design and research processes. The assessment tool was developed through an analysis and synthesis of extant research addressing 3 issues: needs and preferences for older adult technology use for strength building and other exercise [15-18], older adult–specific factors that influence technology acceptance in general [19,20], and barriers and enablers for older adults’ physical activity [16,21,22]. This tool, which we have called the Older Adult Fitness Technology Translation Assessment (discussed in the Methods section), gives us a way to compare our heterogeneous collection of papers against a shared set of older adult fitness technology preference criteria. The use of the word translation emphasizes our approach of focusing on how these technologies will be translated from research into use in practice. We also used this assessment tool to assess gaps in the information collected from the assessment processes to inform best practices.

To our knowledge, no review has compared the design and assessment of existing research on older adult–oriented fitness technology interventions with the known needs and preferences of older adults in terms of technology acceptance and barriers and enablers to physical activity. In undertaking this scoping review, we hypothesized that by reviewing these papers against both their mechanisms of study and these factors for older adult engagement, we would get a clearer picture of the factors that may be leading to the kinds of results the reviews capture.

In the following sections, we first present the Related Work section to situate our work and our main research questions motivating this study. We then present our methodology, including the process of paper selection and assessment criteria. Results, their analysis, and discussion follow.

### Related Work

#### Overview

In this section, we cover the related work that informs the survey methodology and approach. Overall topics discussed are as follows: current reviews in the field, the role of fitness, exercise, and health technology interventions in helping older adults maintain their independence, and a background of older adult–specific technology adoption factors, barriers, and enablers toward physical activity and exercise, and qualitative research on older adult technology preferences.

#### Current Reviews in the Field

As previously introduced, there are as many as 8 reviews in the last 7 years covering older adult fitness and consumer health technologies. Reviews have covered several different technology types including eHealth and mHealth [8,10,12], smart technologies [9], wearables [7,13,14], and VR [11], different health goals including physical activity promotion [7-9,12,14], balance and fall prevention [10,13], and functional mobility [11], and different research focuses including evidence of effectiveness [7-11], adherence [12], and qualitative research on older adult experiences [13,14]. Reviews covering the current state of evidence of effectiveness for older adult eHealth physical activity interventions found mixed evidence of effectiveness overall [8], no difference between smart technology-based interventions and face-to-face physical activity interventions [9], and found evidence of effectiveness for accelerometer-based interventions, but not pedometer-based interventions [7]. The review of older adult eHealth interventions for balance training found potential for evaluating balance assessment and promoting balance training, but without a proper comparison due to differing methodologies [10]. The review of effectiveness for VR-based interventions for functional mobility found evidence for effectiveness compared to conventional treatment, but with low methodological quality [11]. The review of adherence for exercise technology interventions for older adults found high adherence through the first 12 weeks, but with methodological problems, including sample size, inclusion criteria, and follow-up times [12].

Reviews aimed at qualitative feedback from older adults related to wearable technologies for fitness interventions found emergent themes such as the importance of intrinsic motivation, extrinsic motivation, and ease of use [13], and found that most systems focus on supervising rather than supporting older adults and that the existence of co-design was scarce [14]. A summary of the reviews and their major findings is included in Table 1.

#### Importance of Fitness and the Role of Technology for Older Adults

For older adults, research consistently shows that moderate and regular exercise is associated with decreased mortality and the prevention of health conditions such as coronary heart disease, stroke, and type 2 diabetes [3,4,23-26]. Additionally, exercise has been linked to reduced risk of developing dementia and Alzheimer disease [23,27] and linked to increased mental health and well-being. Strength building in particular has been shown to support healthy aging [5,28] and to decrease the likelihood of developing chronic diseases or disabilities later in life [28,29]. As adults age, sarcopenia or the decrease of muscle mass can occur, increasing the likelihood of bone loss (osteoporosis), falls and fractures, and difficulty performing regular daily functions such as walking upstairs or carrying groceries [30-32]. Lower muscle mass is also a risk factor for losing independence in older adults [30,32]. As highlighted in the introduction, the use of technology to address these concerns is a rapidly growing area of research interest. Some of the most researched technologies over the past 10 years include smartphone apps, smartwatches, computer-based interventions, and Microsoft Kinect (Microsoft Corp, Microsoft Kinect Sensor, Microsoft Corp, 2010; see Multimedia Appendix 1 [33-75]).

#### Technology Adoption Research

Technology adoption research aims to better understand what motivates people to adopt certain technologies into their daily lives and to predict which technologies will garner widespread use. Several models have been proposed in the field that outline the factors influencing the public’s willingness to adopt a certain technology. One of the first widely used models for technology adoption, the Technology Acceptance Model (TAM), proposes that adoption is influenced by the potential user’s perceived usefulness and perceived ease of use of the technology [76]. This model has been updated several times with frameworks including TAM 2, unified theory of acceptance and use of technology, TAM 3, and unified theory of acceptance and use of technology extension (UTAUT2). Newer models, such as the UTAUT2, consider factors beyond perceived value and ease of use, including social influence, habit, and price value, and have a higher predictive power than previous models [77].

Despite the existence of updated technology adoption frameworks, most proof of concept and usability studies follow the original TAM model by focusing solely on the ease of use (or usability) of a technology and the user’s enjoyment or perceived value of the product. Examples of the following are commonly used digital usability questionnaires such as the Telehealth Usability Questionnaire [78] and the Health Information Technology Usability Evaluation Scale [79]. Furthermore, for studies on consumer technologies aimed at older adults, the specific needs of older adults and their technology acceptance preferences have been largely overlooked.

#### Older Adult Technology Preferences and Older Adult–Specific Technology Adoption Factors

Research aimed at adapting TAMs specifically for older adults has shown several factors that influence whether an older adult will adopt a new technology. For example, in the studies by Lee [19] and Lee and Coughlin [20], 10 factors are proposed for older adult technology adoption: value, usability, affordability, accessibility, technical support, social support, emotion, independence, experience, and confidence. An overview of the 10 factors included in the model by Lee [19] for older adult technology adoption can be found in Table 2. Another older adult–specific technology adoption reference model by Lindberg and de Troyer [80] identified value, technical support, social support, experience, confidence, usability, emotion, and independence as important user interface design factors for technology acceptance. Qualitative research on older adult preferences related to fitness technology has shown that older adults tend to use traditional, nondigital tools for personal health management, and have mixed reactions to wearables, tracking devices, and smartwatches [81,82]. Major concerns consistent across several studies include privacy concerns, difficulty with onboarding or setup, and cost concerns [80,81]. Privacy concerns were particularly present when discussing health-related wearable devices with older adults unsure of ownership of their data, resistant to the constant monitoring and collection of health data, and with a preference for only collecting data necessary for their specific health concerns [82]. Lastly, identity plays a role in older adults’ willingness to adopt a new health-related technology. For example, many older adults resist adopting assistive technologies if they perceive the device as running contrary to their feelings of independence, self-reliance, and competence [80,81,83]. Thus, it is critical to both the design of the technologies themselves and the verbiage used in the interactions with the user that older adults are met with dignity and not treated as weak or dependent.

**Table 2:** The factors by Lee [19] for older adult–specific technology adoption.

Factor	Description
Value	Perception of the usefulness and potential benefit
Usability	Perception of the user-friendliness and ease of learning
Affordability	Perception of the potential cost savings
Accessibility	Knowledge of the existence and availability in the market
Technical support	Availability and quality of assistance throughout use
Social support	Support from family, peers, and community
Emotion	Perception of the emotional and psychological benefits
Independence	Social visibility, how it makes them look to others
Experience	Relevance to their experiences and interactions
Confidence	Empowerment without anxiety or intimidation

#### Barriers and Enablers to Physical Activity for Older Adults

Beyond research into older adults’ relationship with technology, there is qualitative research on older adults’ needs and preferences as it relates to physical activity and exercise. As we show in our study, this work is rarely referenced by researchers designing and evaluating fitness technology for older adults. Insights from this research have been synthesized as a set of key barriers and enablers to older adults’ physical activity, including factors limiting physical activity and factors encouraging physical activity, respectively. Some common barriers to physical activity for older adults include health conditions [16,21,22], lack of social support [21], inaccessibility [16,21], perceived level of difficulty [22], and fear [16,21,22]. Some common enablers to physical activity include motivation [16,22], enjoyment [22,84], accessibility [16,22], affordability [16], social support [16,21,84], intentionality [16,21,22], and previous or short-term improved health [16,22,84].

#### Lens to View Older Adult Design

As older adult fitness technology research has grown, researchers have begun to reflect on how older adults have been framed with respect to technology, arguing against viewing older adults as homogeneous and in terms of loss of ability or decline in faculty [85,86]. Soro et al [86] noted the prevalence of research aimed to “assist” older adults managing their independence, built on negative assumptions and stereotypes of older adults’ capability. Technologies with this approach are criticized for providing care without a human touch, and can result in disrupted routines, dependency, and isolation [86]. From this work, Soro et al [86] identified two distinct perspectives in the literature: (1) a focus on the technical aspects and (2) a focus on the human perspective, noting that very few works attempt to bridge that gap. Building on that work, Lazar et al [85] proposed the use of diffractive analysis to encourage researchers to include differing perspectives in designing for older adults. Diffractive analysis can help illuminate incorrect assumptions made by researchers and the value of different methodological approaches and perspectives [85]. Co-design or participatory design has also been found to help younger designers fill the knowledge gap and combat assumptions when designing for older adults [87]. Co-design works by establishing an equal relationship between users and designers, and encourages the active participation of users early and often in the design process [87].

### Scoping Review Questions

Building on the above related work, we review the literature by assessing health interventions across what is already known about older adult needs and concerns about fitness technologies and physical activity interventions. We specifically examine health technology interventions intended to help older adults build strength, improve physical fitness, and maintain independence. To assess how well these interventions align with the needs and preferences identified in older adult–specific technology acceptance studies, we have structured a review to answer the following questions:

1. How well does current fitness-related technology research, from any discipline, aimed to support older adults, align with known older adult–specific technology adoption factors, older adult technology preferences, and barriers and enablers to physical activity?
2. How well do current research methodologies evaluate their interventions across the known needs and preferences of older adults?
3. Are the current research methodologies sufficient for understanding whether the technologies will have an impact beyond a research setting?

## Methods

### Study Design

Our review was conducted in compliance with the PRISMA-ScR (Preferred Reporting Items for Systematic Reviews and Meta-Analyses Extension for Scoping Reviews); see Multimedia Appendix 2 for the PRISMA-ScR checklist. The review approach consisted of, first, a search of older adult fitness technology research across the relevant disciplines, second, the selection of papers to be reviewed, third, the summarization of major technology types, intervention types, research goals, and major outcomes of the reviewed papers, fourth, the synthesis of the Older Adult Fitness Technology Translation Assessment tool from older adult’s needs and preferences from older adult–specific technology acceptance, barriers and enablers to older adult physical activity, and qualitative feedback from older adults on fitness technologies research, and, fifth, the comparison of both the design and assessment of the reviewed research papers against the synthesized assessment tool. The search methodology, selection process, summarization of technology types, research goals, and outcomes, synthesis of the Older Adult Fitness Technology Translation Assessment tool, and evaluation process are described in this section.

### Research Paper Selection Process

Work related to health technologies for older adults is present in a variety of different fields, including medicine, engineering, human-computer interaction (HCI), and psychology. Thus, the search strategy targeted the following databases that target these domains to obtain a full interdisciplinary establishment of the evidence in this research topic: (1) ACM Digital Library, for health technology intervention in the field of HCI; IEEE Xplore, for interventions in the field of engineering and computer science; Medline, for interventions in the medical field; and PsycINFO, for interventions in the field of psychology and public health.

The specific search terms in the review were chosen to collect research that related to older adults, used any form of digital health-related technologies, and aimed at building physical fitness or maintaining independence. The following terms and Boolean structure were used to account for the different vocabulary used across fields in this type of work and ensure that selected papers met the 3 requirements of a technology, intended for older adults, and about exercise or strength building: (1) “Older Adults” OR “Elderly”, OR “Elder Adults” OR “Geriatric” OR “Senior,” AND (2) “Digital Health” OR “Technology” OR “Interactive Systems” OR “eHealth” OR “mHealth” AND (3) “Strength Building” OR “Balance Training” OR “Physical Activity” OR “Exercise” OR “Exercise Programs” OR “training program.” Searches were also filtered to only include results from the last 10 years, research papers published in journals, and participants aged 60+ years in Medline. Search terms were adjusted to account for field-specific differences in databases. A full breakdown of the search strategy is included in Multimedia Appendix 3.

From the initial searches, an evaluation on the applicability of the papers was conducted by the research team against the following inclusion criteria:

Studies were included if they:

- Specifically evaluated the impact of technology on physical activity, health, or independence among adults aged 60 years and older, using either quantitative or qualitative methods (eg, interviews or focus groups assessing user experiences).
- Focused specifically on older adults, defined as individuals aged 60 years and older, and directly related to promoting physical activity, building strength, or maintaining independence.
- Mentioned a specific computational technology used in the intervention, such as fitness trackers, mobile health apps, or assistive devices, with the technology being integral to the health intervention.

A detailed review of the full text of each paper meeting the initial inclusion criteria was conducted; aspects such as study design, sample size, intervention methods, and outcomes were evaluated. Studies were excluded if they lacked a clear connection between the technology used and the health intervention outcomes, or if the sample did not include older adults as defined by our criteria. Following this detailed review, a final list of studies meeting all the criteria was compiled.

#### Summary of Older Adult Fitness Technology Types, Research Goals, and Major Outcomes

The reviewed papers were summarized into major themes, including technology types, intervention types, and outcomes of existing older adult fitness technologies across the reviewed domains. Research papers were categorized based on the overall and immediate health goal, research goal, study target, number of participants, study length, presence of a control group, presence of older adult–specific design methodologies, presence of co-design in the design process, number of interaction components, and major outcomes to identify emergent themes and provide a summarization of the current state of research. Results were summarized and coded only using information available to reviewers in the research papers themselves.

#### Synthesis of the Older Adult Fitness Technology Translation Assessment

##### Overview

Using existing research from different associated areas within the field, we synthesized 6 criteria to evaluate the current older adult fitness technology interventions. As explored in the related works sections, an abundance of research has been conducted to better understand older adults’ needs and preferences when it comes to both technology and physical activity. Examples of these spaces that were used in the synthesis of the evaluation criteria include older adult–specific technology adoption factors, qualitative research of older adults’ health and technology preferences, and barriers and enablers to physical activity for older adults. As older adult fitness technology interventions require older adults to both interact with technology and participate in some form of physical activity interventions, it is critical to consider older adult preferences related to both areas, though, as will be evidenced, many of the important factors emerge across fields.

The 6 criteria are as follows:

##### Compatibility With Lifestyle

The work by Lee [19] on older adult–specific technology acceptance coined the term “compatibility” to refer to the concept that “if a technology does not seamlessly fit into older adults’ life patterns or mental models, they are less likely to adopt it.” From this work, older adults identified the need for technologies to fit into daily schedules and physical spaces as well as have conceptual compatibility with their culture and values. Similarly, the work from Naseri et al [21] on barriers and enablers for older adult engagement in fall prevention activities, physical opportunity, and environmental context was identified as a barrier for participation. Emergent themes from this barrier included a lack of access to supervision support, suitable environments, and proper equipment. Naseri et al [21] include a specific example showing how a lack of compatibility can act as a barrier to a participant’s response, referencing needing to go to the gym for exercise, “when I’m cold I just stay in bed and don’t exercise.” In the work by Thøgersen-Ntoumani et al [22] on barriers and enablers to intermittent lifestyle physical activity, both “convenience” and “reframing physical activity” were identified as enablers. Older adults were more likely to do physical activity if it could be incorporated into their daily lives or they could do it while doing other meaningful activities, such as playing with children and cleaning the house. Older adults identified that they disliked going to the gym and doing structured exercise, which requires planning; thus, reframing everyday activities as exercise was an important enabler.

With this context, we chose the term “compatibility with lifestyle” to refer to how well a fitness technology intervention fits into the potential user’s overall lifestyle, including daily routines, habits, preferences, and values.

##### Similarity to Past Experience

The UTAUT2 by Venkatesh et al [88] identifies users’ previous “experiences” as an important factor for influencing technology acceptance. For older adult–specific technology acceptance, Lee and Coughlin [20] identified “experience” as an important technology acceptance factor for older adults specifically, referring to how relevant the technology is with their prior experiences and interactions. Technologies that are perceived to be more familiar to older adults or that build on prior knowledge are more easily adopted. In addition to the technology design itself, the training methods and education are influenced by this factor as well. Building off the older adult–specific technology acceptance work by Lee [19], the reference model by Lindberg and De Troyer [80] for older adult–specific technology acceptance identifies that technologies should ideally resemble the user’s prior experience with other systems. While this is challenging in practice due to the diverse experiences of users, similarity with past experiences can be addressed by allowing users to personalize their interactions. In the work by Pang et al [81] on older adult–specific technology acceptance and learning preferences, the importance of including flexible learning materials and instruction manuals was explored, and a preference by older adults for “augmenting current technologies” rather than general-purpose wearables was noted.

With this context, we chose the term “similarity with past experience” to refer to how similar the technologies older adults interact with are to their previous experiences and how well training, education, and personalization are incorporated into the intervention to prevent difficulties in uptake.

##### Dignity and Independence

In the older adult–specific technology acceptance reference model by Lindberg and De Troyer [80], “independence” is identified as a key factor for technology acceptance. In their work, Lindberg and De Troyer warn that “the language used in an application should avoid stigmatizing the user” and “older people who feel vulnerable or dependent on others may easily perceive an application as a threat to their independence or [feeling] patronized by the application.” The work by Astell et al [89] on older adults’ assistive technology preferences found that identity is a major factor for acceptance. Older adults tend to resist the use of assistive technologies that constantly remind them of their old age and the negative connotations associated with that, such as conflation of “oldness” and “disability” with “helplessness,” “dependence,” and “incompetence.” For example, 1 participant in this work stated when referencing an assistive robot, “It must be for people who are very handicapped. It’s not for me… It makes me think my life is terminated. I’d rather die than live with a robot.” Furthermore, being stigmatized or discriminated against was a common fear for many participants and strongly impacted older adults’ willingness to adopt assistive technologies. Devices that could stigmatize older adults as “different,” “lonely,” “frail,” “dependent,” or “old” were not popular. The work by Astell et al [89] also found that independence and control were key contributors toward acceptance.

With that context, we chose the term “dignity and independence” to refer to whether the health intervention, user interface, verbiage, and technology itself promote a feeling of self-sufficiency and independence in its users, or does it promote stigmatization and a feeling of dependency on technology.

##### Privacy Concerns

Pang et al [81] identify privacy as a major concern for accepting health technologies, finding that older adults were reluctant to compromise privacy for general wellness tracking. Some older adults expressed a preference for only tracking data directly relevant to their health condition, that is, blood pressure measurements, over perceived frivolous extras such as tracking distances and routes. In the work by Zhang and Shahriar [82] on issues related to older adult wearable health care technologies, data privacy was identified as a widespread concern. Many older adults were unsure of the data storage and privacy policies of the wearables. Furthermore, Zhang and Shahriar urged for more data privacy education and prompt manufacturers to clearly state how data is used and allow users the ability to opt out of providing it. Older adults were also found to be resistant to the over-collection and misuse of data, particularly related to wearables that require continuous use. Astell et al [89] raised the concern of privacy versus pragmatics with assistive technologies aimed at older adults, that is, the perceived “trade off” between personal privacy and the potential benefits of technologies. Technologies such as wireless sensor networks, bed occupancy sensors, and fall detection monitors were all met with privacy concerns. Older adults were also reluctant to adopt monitoring or surveillance technologies, fearing someone “always watching” or “spying” on them.

With this context, we chose the term “privacy concerns” to refer to how well the user’s privacy concerns related to personal information and data are respected, with a preference for technology systems that include clear data storage protocols and privacy policies, the ability to opt-out of certain data collection, and limit the amount of data collected to only the directly relevant data necessary.

##### Social Support

Lee and Coughlin [20], and Lee [19] identify social connection and support as a key factor for older adult–specific technology adoption**.** Older adults were more willing to adopt a technology if peers in their social circle (family, friends, and community members) also used the technology. The use of peer leaders, well-connected early adopters, and technology champions was critical. Furthermore, older adults may view technology as a potential threat to decreased social connectivity and emotional contact, hindering adoption. Technologies that incorporate a way to easily connect with friends and family can become more attractive to older adults. In the work by Naseri et al [21] on barriers and enablers to older adult physical activity, social support also played a key role. Access to social support, family assistance, partner participation, family, and trusted advisors were all identified as enablers. Furthermore, positive models of behavior from peers can act as an enabler; that is, if their friends, family, and neighbors are all exercising and having positive experiences doing it, older adults are more likely to join. Thøgersen-Ntoumani et al [22] found social engagement to be an important enabler toward older adult physical activity through the normalization of taking the active option. An example of this is if 1 participant took the stairs instead of the lift, it encouraged other people to take the stairs as well. In the work by Pang et al [81] on learning preferences, after initial independent problem-solving, older adults tended to prefer to turn to family or peers for help with technologies. Incorporating a social network between them could help older adults learn from each other and foster independence.

With this context, we chose the term “social support” to refer to the ability of the health intervention or technology to encourage or support social connection.

##### Emotion

In the UTAUT2 by Venkatesh et al [88], “hedonic motivation” was identified as an important factor toward technology acceptance, referring to the fun and pleasure derived from using a technology. Lee and Coughlin [20] identified “emotion” as a key factor to older adult–specific technology acceptance, referring to the perceived emotional and affective benefits. Naseri et al [21] identified several emotional aspects as key enablers to older adult physical activity, including motivation, confidence, and novelty. Additionally, Thøgersen-Ntoumani et al [22] found several emotional aspects to be enablers such as gamification, sense of achievement, and personally salient rewards.

With this context, we chose the term “emotion” to refer to the ability for the intervention or technology to be motivating, fun, or emotionally stimulating.

In our evaluation, we also considered the short- and long-term outcomes from the research studies to examine both how well the health technology affected positive change for the participant and how well the outcome metrics in the research study aligned with the stated objectives. Outcomes were evaluated based on the results in the study and the stated use case of the intervention found in the respective research papers.

##### Short-Term Outcomes

These refer to the specific outcomes related to the main objective of the study immediately following the health intervention. In this case, outcomes can include behavioral changes (eg, physical activity increase) or health markers (eg, strength measures).

##### Long-Term Outcomes

These refer to the specific outcomes related to the main objective of the study in the long term. This can be with continued use of the health intervention or technology or for a specific period of time after the intervention or technology is no longer in use. The implications of this difference will be discussed further in the Discussion section of the paper.

#### Older Adult Fitness Technology Translation Assessment Process

To evaluate the 43 papers included in this review consistently, a structured rubric was developed, shown in Multimedia Appendix 4. For each category, the rubric identified the specific qualities of health intervention that would best address their respective concerns and specified what interventions would look like. The rubric included a range from 1 to 5, with 1 being a technological intervention that is most in conflict with the given category and 5 being most in harmony with the given category. For example, using the compatibility with lifestyle category, an intervention that seamlessly integrates into a potential user’s current daily life patterns without much additional effort would be given a score of 5, and an intervention that requires a lot of extra time and effort to integrate into the potential user’s lifestyle would be given a score of 1.

The scoring process consisted of 5 reviewers, who individually assigned a score to each paper within every category based on how well the paper met the established criteria. To ensure that every paper was evaluated from at least 2 different perspectives, each paper was assigned to 2 independent reviewers using a dual-review approach. On occasions where the 2 reviewers disagreed with their assessment by a score of 2 or more, all 5 reviewers reviewed the papers to resolve the disagreement through consensus. Interrater reliability was reported using a quadratic weighted Cohen κ statistic [90]. Results from the evaluation were summarized by primary technologies, interaction types, and goals of the interventions to better understand how different types of interventions aligned with the known needs and preferences of older adults and identify potential trends.

## Results

### Overview

Included in this review are (1) overall themes and categories of research in this space; (2) a summary of the common technologies, interaction types, and goals; (3) overall takeaways from intervention types and research methodologies; and (4) a comparison of the health interventions with known preferences of older adults from older adult–specific technology adoption work.

**Figure 1:**
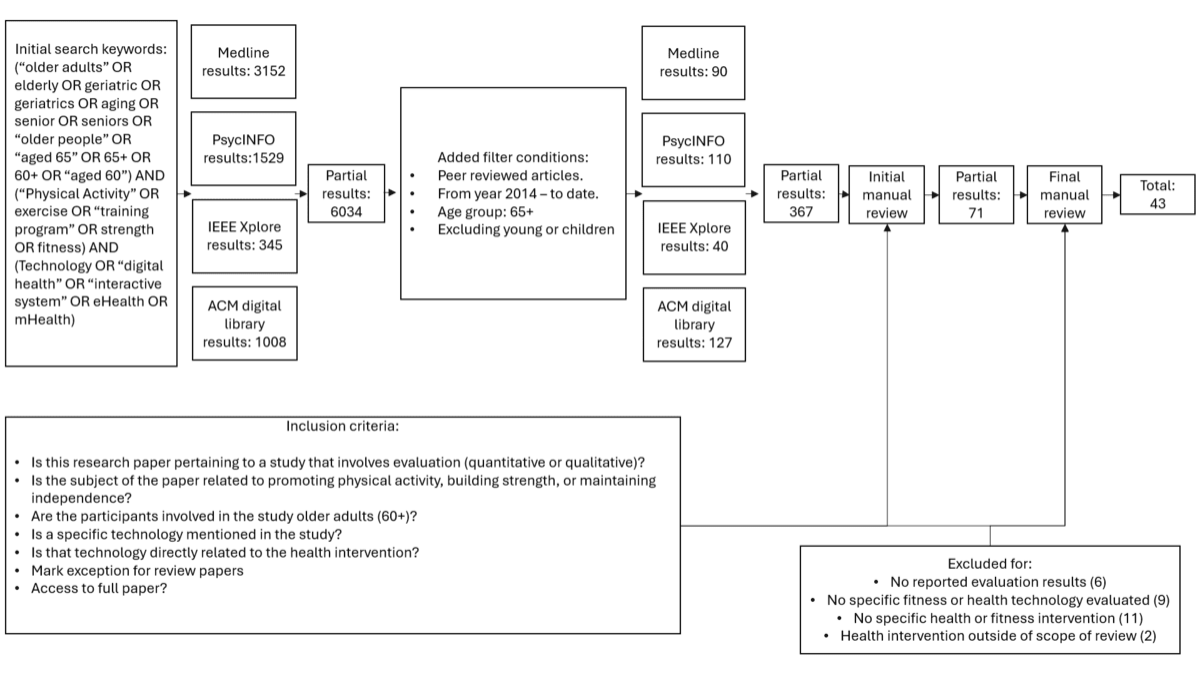
Flowchart of the paper selection process in the review.

**Figure 2:**
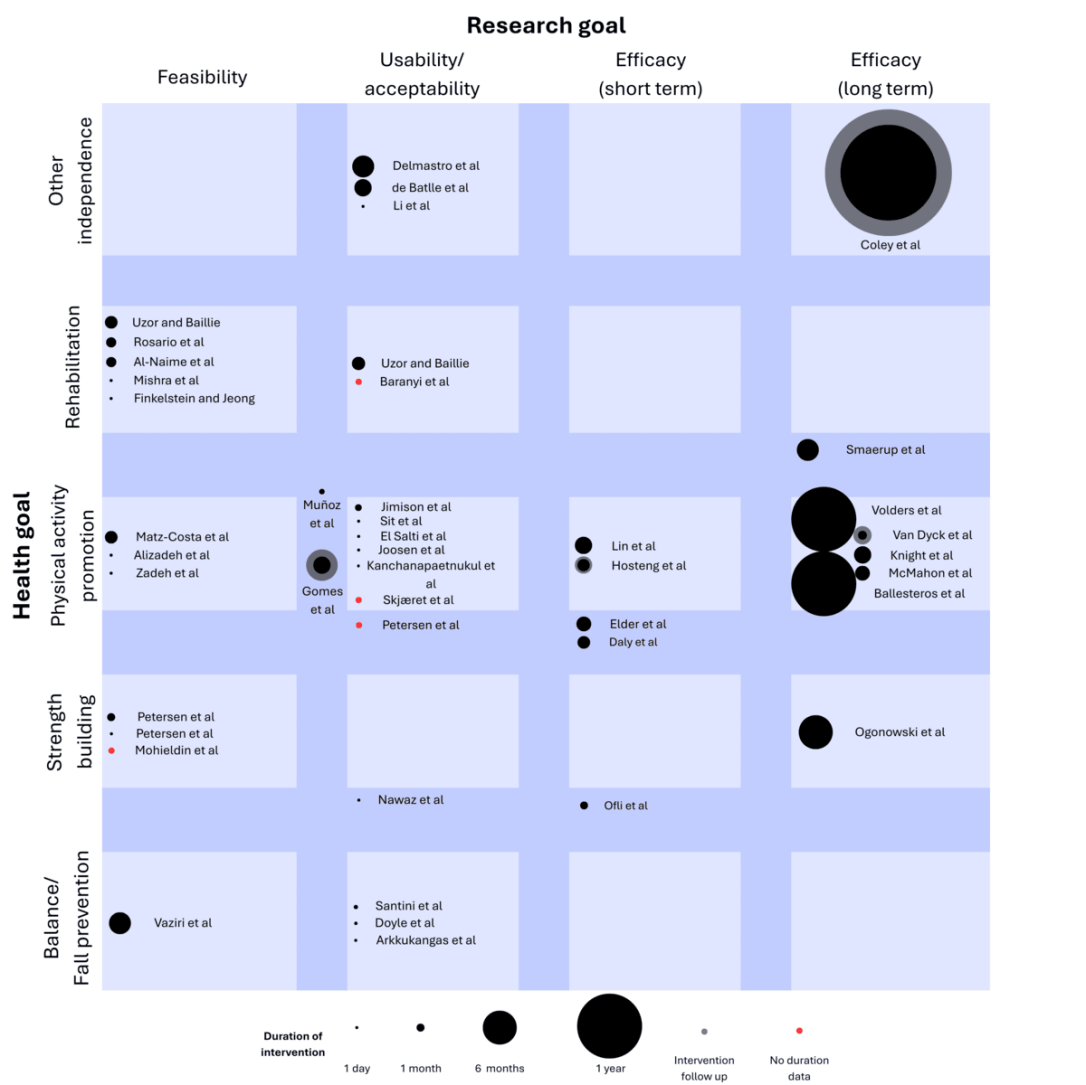
Categorization plot of the research goals, health goals, and intervention duration for the research papers included in the review. Research goals were categorized into feasibility, usability, and acceptability, short-term efficacy, and long-term efficacy studies based on self-identification. Health goals were categorized into strength building, physical activity promotion, rehabilitation, and other independence. Studies that fit into more than 1 category are shown at the boundaries. Bullet size is proportional to intervention length. Specific values for each intervention are found in Multimedia Appendix 1 [48-90].

The 43 research studies were summarized by the journal they were published in, technological system, health goal, research goal, study target, number of participants, study duration, whether there was a control condition, and finally, the major outcomes presented in the work. A flowchart of the full selection process is shown in Figure 1. Results from this process are shown in Figure 2.

### Summary of the Reviewed Research Papers

Overall, 16 of the review papers were submitted to medical journals, 15 were submitted to engineering journals, 7 were submitted to HCI or computing journals, 3 were submitted to public health journals, and 2 were submitted to a cross publication between computing and engineering journals. The research focus of the papers was usability, feasibility, and acceptability for 27 of the papers, studies of effects for 14, and design for 2 of the articles. A total of 13 of the 43 research papers were controlled studies. Further, 22 of the research papers were of studies longer than 4 weeks, 4 lasted between 1 and 4 weeks, 13 lasted only a single day, and 4 did not specify a study duration.

### Summary of Fitness Technology Intervention Types

#### Overview

The primary user interaction technologies used in the selected health interventions include six different types: (1) 12 for Microsoft Kinect; (2) 15 for wearables such as smart watches, heart rate monitors, etc; (3) 18 for mHealth apps (phone or computer-based); (4) 7 for smart fitness equipment such as resistance bands, exercise bikes, etc; (5) 3 for augmented or virtual reality; and (6) 2 for website.

Six common interaction types were found in the selected interventions: (1) 13 for game, (2) 18 for self-tracking, (3) 2 for physician-tracking, (4) 28 for personalized health advice, (5) 1 for competition, and (6) 4 for planning.

Despite disparate overall objectives, including general well-being promotion, sarcopenia treatment, fall reduction, and rehabilitation, to achieve those objectives, four common immediate health goals were observed in the selected interventions: (1) 10 for building strength, (2) 33 for increasing physical activity, (3) 9 for training balance, and (4) 5 for training mental fitness.

A comprehensive list of the stated research and health goals of the selected interventions, study target, number of participants, and study duration is included in Multimedia Appendix 1. Reported outcomes included behavioral change outcomes (eg, minutes of exercise), health outcomes (eg, blood pressure), technological functioning (eg, accuracy), and user feedback on usability and usefulness (both quantitative and qualitative).

#### Presence of Older Adult–Specific Design Methodologies

Before assessing the research papers based on the older adult design needs and preferences synthesized in this work, research studies were reviewed for the presence of cited older adult–specific design research in their methodologies to obtain a basic understanding of how many of these works were being used to inform technology design in the field (Multimedia Appendix 5 [33,35-48,50-75,91-111]). Of the 43 papers reviewed, 16 explicitly reference research on older adult design factors. Among these, 11 cite such factors in their background and related works section, 10 cite them specifically as part of their design approach, and 4 incorporate them directly into their methodology. Of the 16 research papers that explicitly reference older adult design works, reviewers scored 6 of the corresponding intervention designs as directly consistent with the stated requirements from the cited older adult design works, 1 as mostly consistent, 3 as somewhat consistent, 1 as directly contradictory, and 5 as not having enough information to make an assessment. A summary of the cited older adult design works can be found in Multimedia Appendix 1.

#### Overview of Findings for the Assessment of Older Adults’ Needs and Preferences

On average, the interventions scored 2.93 (SD 0.86) on compatibility with lifestyle, 3.10 (SD 0.74) on similarity to experience, 3.49 (SD 0.64) on dignity and independence, 3.17 (SD 0.86) on privacy concerns, 3.74 (SD 0.81) on short-term outcomes, 2.75 (SD 1.21) on long-term outcomes, 2.79 (SD 0.88) on social support, and 3.17 (SD 1.19) on emotion. Distribution of older adult–specific technology adoption scores for the 43 selected interventions is shown in Tables 3-5. The weighted κ scores for compatibility with lifestyle were 0.448, similarity with past experience was 0.301, dignity and independence were 0.145, privacy was 0.204, social support was 0.299, and emotion was 0.421. For qualitative classification tasks, some disagreement is expected and in some cases important, and lower-than-normal weighted κ scores are common [112]. However, due to the relatively low κ scores overall, the researchers met to review major disagreements and come to a consensus, a practice consistent with the recommendations on reliability and interrater reliability for HCI practice [112]. Major disagreements were flagged if the scores differed by more than 2 or if there was any disagreement on whether enough information was present to review the factors. A total of 22 disagreements of 344 datapoints from 17 research papers were resolved via consensus. Notes on the reasons for disagreement and consensus rationales are included in Multimedia Appendix 6.

Beyond overall scores, an assessment of the scores across primary interaction technologies, interaction types, and common goals was conducted.

**Table 3:** The average scores of the six older adult–specific technology adoption factors across the six common critical technologies found in the survey: (1) mHealth (apps), (2) wearables, (3) Microsoft Kinect, (4) smart fitness equipment, (5) AR/VR^a^, and (6) website.

Critical technology	Number	Compatibility, mean (SD)	Similarity, mean (SD)	D and I^b^, mean (SD)	Privacy, mean (SD)	Social, mean (SD)	Emotion, mean (SD)
mHealth (apps)	18	3.25 (0.96)	3.33 (0.62)	3.56 (0.66)	3.31 (0.99)	2.97 (0.90)	2.83 (1.00)
Wearables	15	3.40 (0.83)	3.47 (0.83)	3.47 (0.69)	2.73 (0.73)	2.83 (1.19)	2.70 (1.03)
Microsoft Kinect	12	2.46 (0.66)	2.63 (0.48)	3.46 (0.75)	3.17 (0.75)	2.54 (0.58)	4.00 (0.83)
Smart fitness equipment	7	2.86 (0.48)	3.07 (0.67)	3.71 (0.49)	3.29 (0.99)	3.07 (0.89)	2.64 (1.35)
AR/VR	3	2.33 (0.76)	2.50 (0.50)	3.50 (0.50)	3.50 (0.50)	3.00 (0.87)	4.67 (0.29)
Website	2	3.75 (0.35)	4.75 (0.35)	2.75 (0.35)	3.00 (0.71)	2.00 (0.00)	1.75 (0.35)
Total	43	2.93 (0.86)	3.10 (0.74)	3.49 (0.64)	3.17 (0.86)	2.75 (0.88)	3.17 (1.19)

^a^AR/VR: virtual and augmented reality.

^b^D and I: dignity and independence.

**Table 4:** The average scores of the six older adult–specific technology adoption factors across the six common critical interactions found in the survey: (1) personalized health advice, (2) self-tracking, (3) game, (4) planning, (5) physician-tracking, and (6) competition.

Critical interaction	Number	Compatibility, mean (SD)	Similarity, mean (SD)	D and I^a^, mean (SD)	Privacy, mean (SD)	Social, mean (SD)	Emotion, mean (SD)
Personalized health advice	28	3.18 (0.85)	3.29 (0.76)	3.45 (0.67)	3.20 (0.93)	2.71 (0.97)	2.77 (0.94)
Self-tracking	18	3.08 (0.97)	3.28 (0.65)	3.58 (0.81)	3.28 (0.93)	2.72 (0.77)	2.81 (1.21)
Game	13	2.38 (0.71)	2.73 (0.60)	3.58 (0.64)	3.00 (0.71)	2.92 (0.76)	4.38 (0.51)
Planning	4	4.00 (1.08)	4.25 (0.65)	3.63 (0.75)	3.13 (0.48)	2.75 (0.87)	2.25 (0.65)
Physician-tracking	2	3.25 (0.35)	3.50 (0)	4.00 (0)	2.75 (0.35)	2.75 (0.35)	4.00 (0)
Competition	1	4.00 (N/A^b^)	4.00 (N/A)	4.50 (N/A)	2.00 (N/A)	4.00 (N/A)	3.50 (N/A)
Total	43	2.93 (0.86)	3.10 (0.74)	3.49 (0.64)	3.17 (0.86)	2.75 (0.88)	3.17 (1.19)

^a^D and I: dignity and independence.

^b^N/A: not applicable.

**Table 5:** The average scores of the six older adult–specific technology adoption factors across the four common intervention goals found in the survey: (1) strength building, (2) physical activity promotion, (3) balance training, and (4) mental fitness training.

Intervention goal	Number	Compatibility, mean (SD)	Similarity, mean (SD)	D and I^a^, mean (SD)	Privacy, mean (SD)	Social, mean (SD)	Emotion, mean (SD)
Physical activity	33	3.08 (0.88)	3.17 (0.76)	3.42 (0.64)	3.20 (0.94)	2.82 (0.97)	3.09 (1.14)
Strength	10	2.55 (0.72)	2.90 (0.57)	3.55 (0.83)	3.10 (0.77)	2.75 (0.59)	3.30 (1.48)
Balance	9	2.33 (0.75)	2.78 (0.57)	3.50 (0.83)	2.72 (0.62)	2.50 (0.79)	3.94 (0.92)
Mental fitness	5	3.20 (1.35)	3.40 (0.96)	3.20 (0.91)	3.20 (0.57)	2.40 (0.22)	3.40 (1.34)
Total	43	2.93 (0.86)	3.10 (0.74)	3.49 (0.64)	3.17 (0.86)	2.75 (0.88)	3.17 (1.19)

^a^D and I: dignity and independence.

#### Results by Primary Interaction Technologies

The average scores for the most common technologies used, apps, wearables, and Microsoft Kinect-based systems were 3.20 (SD 0.42), 3.07 (SD 0.51), and 3.32 (SD 0.76), respectively. Between the 3 most common technology types, wearables scored highest in compatibility with lifestyle (mean 3.40, SD 0.83) and similarity with past experience (mean 3.47, SD 0.83), apps scored highest in dignity and independence (mean 3.56, SD 0.66), privacy concerns (mean 3.31, SD 0.99), short-term outcomes (mean 3.82, SD 2.01), and social support (2.97, SD 0.90), and Microsoft Kinect scored highest in long-term outcomes (mean 4.50, SD not applicable) and emotion (mean 4.00, SD 0.83). Full results are included in Table 3.

#### Results by Interaction Types

The average scores for the most common interaction approaches, personalized health advice, self-tracking, and exergames were 3.12 (SD 0.34), 3.13 (SD 0.44), and 3.22 (SD 0.70), respectively. Between the 3 most common interaction approaches, personalized health advice scored highest in compatibility with lifestyle (mean 3.18, SD 0.85) and similarity with past experience (mean 3.29, SD 0.76 ), self-tracking scored highest in privacy concerns (mean 3.28, SD 0.93), and exergames scored highest in short-term outcomes (mean 4.00, SD 2.14), social support (mean 2.92, SD 0.76), and emotion (4.38, 0.51). For dignity and independence, both self-tracking and exergames tied with a score of 3.58 (SD 0.81, SD 0.64). For long-term outcomes, both personalized health advice and exergames tied with a score of 2.75 (SD 0.65, SD 2.47). Full results are included in Table 4.

#### Results by Common Goals

The average scores for the most common health goals, increase physical activity, train balance, and build strength were 3.10 (SD 0.37), 3.32 (SD 0.85), and 3.31 (SD 0.63), respectively. Between the 3 most common health goals, interventions aimed at increasing physical activity scored highest in compatibility with lifestyle (mean 3.08, SD 0.88), similarity to experience (mean 3.17, SD 0.76), and privacy concerns (mean 3.20, SD 0.94), training balance scored highest in short-term outcomes (mean 4.30, SD 2.29) and emotion (mean 3.94, SD 0.92), and building strength scored highest in dignity and independence (mean 3.55, SD 0.83). For long-term outcomes, both interventions aimed at building strength and training balance tied with a score of 4.5 (SD not applicable, SD not applicable). Full results are included in Table 5.

#### How Long-Term Outcomes Are Assessed

Long-term outcomes are essential for understanding the sustained effectiveness of interventions, especially those designed to improve physical activity or independence in older adults over time. Reviewers were asked to only consider either behavioral change outcomes (eg, minutes spent exercising) or health outcomes (eg, blood pressure) as other outcomes, such as usability and usefulness, would be assessed in other technology adoption categories. Only 6 of the 43 research papers reported any long-term outcomes of the interventions, despite many of the interventions being intended for long-term use in practice. Reviewers categorized the reasons for the missing long-term outcomes in three categories: (1) the intended use of the health intervention is short-term (eg, rehabilitation interventions), (2) the research was not yet at the stage where long-term studies were feasible, and (3) the reviewers suggested that long-term outcomes should have been considered. Reviewers exempted research studies that recorded short-term health or behavioral change outcomes from category 2, though they are aware that in many cases, the time and resources needed to conduct a long-term study are prohibitive. Scores are only meant to show the potential areas for further study in the field. Of the 37 research papers that did not include long-term outcomes, 8 were meant for short-term applications, 16 were not yet at the stage of development, and 16 could have benefited from recording long-term outcomes. These scores not only reflect the current effectiveness of various technologies, interaction methods, and health goals but also highlight areas that need further research and refinement to better support older adults’ health outcomes. For example, while Microsoft Kinect shows strong long-term outcomes, its impact on privacy concerns and lifestyle compatibility could be explored in future studies.

### Complexity

Interventions were also assessed for the number of technological components necessary for older adults to interact with the fitness interventions, an approximate measure of complexity, and a portion of similarity and compatibility factors. Roughly half, 21 of 43, of the interventions required older adults to interact with 1 or 2 technological components, and 38 of the 43 interventions required older adults to interact with 4 or fewer technological components, as assessed by reviewers. One health intervention required older adults with 10 different identified technologies, and another with 12 [66,71].

### Co-Design

An additional investigation into the presence of co-design, a design methodology shown to reduce assumptions and better align design choices with the preferences of potential users [87], found that only 13 of the 43 papers mentioned any use of co-design. The types of co-design found include focus groups, user experience interviews, and specified user-centric design processes.

## Discussion

### Principal Findings

Overall, the results of this scoping review illuminate 3 main opportunities for growth in the field, better alignment between the studied needs of older adults around technology interventions and the current technology interventions for research around technology for older adult fitness, better translations of the findings of older adult design work to the designs in practice, and more explicit usage of older adult–specific considerations in the research methodologies for this growing area of research.

### Alignment Between Expressed Older Adult Needs and Current Technology Interventions

From the Older Adult Fitness Technology Translation Assessment, none of the interventions scored a 3 or above on all 8 factors as assessed by our reviewers, suggesting that there was a least 1 easily identified older adult–specific translation problem for every single one of the included interventions as compared to the understood needs and preferences of older adults. The overall middling averages across all the papers considered, and the lack of a consistent pattern across technologies, interactions, and goals between interventions, point to differing strengths and widespread weaknesses. The results from our assessment also point to a need to learn from other intervention designs and interdisciplinary works to better incorporate older adult–specific needs into the designs of older adult fitness technology interventions. The alignment of new designs with the identified factors around older adult preferences has the potential to afford better effects and increase potential for uptake with minimal changes to designs. For example, concerns around compatibility, similarity, and privacy include the space and cost requirements of technologies such as Microsoft Kinect, the potential daily life disruption of strictly regimented exercise plans, the uptake concerns around complex and unapproachable technology systems, and the unease with systems that require 24/7 monitoring, such as fitness wearables. The lack of alignment found in this review was more tied to the contextual details of the overall fitness technology system design rather than the specific technology types themselves. The research team recommends caution when adding additional secondary features, technologies, or data collection that is not directly tied to the specific older adult health goal, as it can run the risk of adding complexity of use for older adults and may introduce resistance based on privacy concerns. Additionally, we recommend a focus on minimal disruption and flexibility of use, including independent and open intervention plans that are more easily incorporated into routines and simple technology interactions with minimal space requirements and easy uptake. Small changes to intervention design could make a significant difference in the experience for older adults. For example, a wearable and app-based intervention to increase step count could, instead of requiring 24/7 monitoring, tie daily goals to only additional “exercise steps” and emphasize to older adults that they only need to wear it when they feel comfortable doing so. Additionally, a Microsoft Kinect-based exercise program could instead opt for a follow-along video-based intervention. What may be lost in interaction and progress monitoring may be gained through the reduction in space requirements and the overall simplicity. In summary, almost any technology or approach can work if it fits with and respects the older adult preference criteria. Drawing on awareness of older adult technology concerns, potential translation problems in the future of even the most promising interventions could have been identified and adjusted for early in the design process.

### Translation From Cited Older Adult–Specific Design Works to Design in Practice

To better understand the reason for the middling results, reviewers looked to the presence of referenced older adult–specific design work in the reviewed research papers. If very few of the research papers referenced older adult–specific design work, then it could point to a lack of awareness in the field as the reason for the mixed results. Of the 43 research papers, only 16 explicitly cited older adult–specific design works, and only 11 directly referenced those works as part of the fitness technology design. Furthermore, in all the referenced older adult–specific design works, none of them encompassed all the considerations outlined in the Older Adult–Specific Technology Translation Assessment, with 5 of the research only citing works relating to the physical limitations of older adults. This points to inconsistent consideration of the full scope of older adults’ needs and preferences related to fitness technologies.

However, for the research papers that did cite older adult–specific design works, reviewers also found potential evidence of a lack of translation of the outcomes from the cited works and the design choices of the reviewed papers. Only 6 of the reviewed fitness technology interventions fully aligned with the stated outcomes in the older adult design works. An example of this disconnect between the use of older adult–specific design research and the proposed interventions is illustrated in the work of Ogonowski et al [43], chosen as an exemplar because it follows the design approach mostly closely related to what is being proposed as effective in this review. That is, it cited older adult–specific design considerations in the background, design, and methodology, and used participatory research or co-design with older adults. In this example, viewing only the older adult design work cited as forming the system requirements for their fitness technology intervention, a disconnect was found. The cited older adult–specific design work by Meurer and Wieching [100] found a preference against the online social networks and menus with “lots of other accessories and other extras,” and a preference for enabling in-person social connections and simple, easy-to-use technology interactions. Despite those identified preferences, the resulting technology included a social media platform for participants in which participants could meet other users virtually and post training results, news, questions, and suggestions [43]. When comparing it to the known research surrounding the needs and preferences of older adults using the Older Adult Fitness Technology Translation Assessment tool, compatibility concerns were raised over the space requirements of the technology, and privacy concerns were raised over the continuous monitoring of older adults through the senior mobility monitor.

### Ethical Need for More Older Adult–Specific Considerations in Research Methodologies

In conducting the scoping review, many of the questions from the Older Adult Fitness Technology Translation Assessment remained unanswered from the published outcomes of the reviewed studies, such as how well the technologies fit into older adults’ routines, how effective the interventions were over the long term, and how effective the interventions were outside of a research setting. The immediate implication of this is that it was hard to predict how well the technology interventions would translate to older adult use in practice. For technology interventions aimed to be used in older adults’ homes, in community centers, as part of public health initiatives, and in medical interventions, ethically, the implications of use in practice should be considered, including older adult–specific needs and preferences to avoid wasting funding and increase the likelihood of success beyond research environments. The reported outcomes from the reviewed studies include strength gains, balance improvements, increased minutes of physical activity or daily steps, perceived usefulness and utility, usability, functionality, and general qualitative feedback from older adults. However practical considerations for older adults remain unanswered, such as how well does the proposed technology intervention fit into older adults’ daily lives, routines, and space at home, do older adults have any privacy concerns when using the intervention, how similar is the interaction to something they have performed before, and does the intervention promote a feeling of dignity and independence for older adults.

Asking these questions can help progress the field by building insights for how older adults feel about different fitness technologies and intervention types, and supporting the overall older adult–specific fitness technology factors with direct outcomes from the field. For example, which types of social engagement systems work best, which intervention types are more likely to fit into routines, and which technologies are older adults more willing to engage with. Implementing explicit inclusion of older adult–specific factors into the assessment methodologies of interventions in the field could help to address this gap and avoid potential pitfalls that may otherwise only be found once used in practice.

### Summary: Potential Value of Explicitly Considering Older Adult–Specific Factors

This scoping review has considered papers across a range of fields that all have in common presenting novel designs of or novel use of digital technologies to support building or sustaining fitness for older adults. To quantify factors across these papers that may be affecting the efficacy of this research as modeled across the other cited reviews in this domain, we have organized the included papers against several features, such as types of technology used, types of evaluation carried out. We also developed novel assessment criteria based on research literature related to older adult technology acceptance and older adult physical activity uptake. We used the resulting 6 factors as a further way to assess design efficacy.

Overall, as highlighted in the analysis of the results above, while there is awareness of design-specific guidance for older adult technology uptake, there is strong evidence of a disconnect between known older adult preferences and the design and assessment of proposed older adult fitness technologies in the field. This evidence was present across all of the reviewed research domains, including medical, engineering, and HCI.

As we underscore in point 3 in the discussion, this disconnection has implications for the development and delivery of ethical research. It means that participants’ time may be wasted, not least among potentially more vulnerable participant populations of older adults. Considering the needs or preferences of a particular demographic is standard practice to inform design. That there is both a rich literature to draw on around older adult technology adoption and around barriers to fitness uptake, and that this work is largely absent from work around older adult technology adoption for fitness, has implications for the appropriate use of research funding, when known affordances and constraints are not explicitly considered.

### Conclusions

Overall, this review contributes the following, an analysis of the common strategies of older adult fitness technology interventions and the strengths and weaknesses in the field and a synthesis of the needs and preferences of older adults from older adult technology acceptance research, barriers and enablers to older adult physical activity, and qualitative data from older adult fitness technology research into an assessment tool called the Older Adult Fitness Technology Translation Assessment Tool.

We proposed a novel rubric to assess across the 6 older adult–specific criteria how well the design and assessment of the presented older adult fitness technology systems align with the known needs and preferences of older adults in engaging with fitness technologies. Our goal has been to create a mechanism that enables very different technologies and approaches to still be compared against critical, meaningful criteria. This analysis does show an overall inconsistent consideration of any of our 6 older adult–sensitive criteria. This finding suggests that if not causation, then a likely strong correlation exists between the types of middling effects captured in the existing review papers and systemic limited attention to older adult–sensitive design and evaluation criteria. Considering the topic area is to support older adult adoption, via technology, of sustainable fitness practices, this lack of attention to well-established older adult technology dynamics is surprising. In general, the results of this review suggest that there is a significant opportunity for improving and revisiting how to best use the potential of these technologies to impact older adults’ daily lives and well-being positively by starting the design process with the known preferences and constraints of older adults and adjusting our assessment processes to explicitly consider older adult–specific factors beyond usefulness and usability.

A limitation for our analysis is the lack of detailed information on the older adult interaction with the technologies and the lack of outcome metrics that are directly related to the factors included in the Older Adult Fitness Technology Translation Assessment tool. Thus, reviewers needed to use their best judgment from the information provided in the published research papers. Second, due to the cross-field nature of these technologies, the keywords used in the search strategy may not have accounted for all the possible older adult fitness technologies across all the relevant fields. Future work is planned to validate the Older Adult Fitness Technology Translation Assessment tool for novel older adult fitness technology design and assessment, including developing and testing a design framework based on the Older Adult Fitness Technology Translation Assessment tool with a novel fitness technology prototype. The assessment will include an initial multistage iterative trial and a follow-up 6-month randomized controlled trial to assess the effects of using the framework, measuring adherence, health outcomes, and older adult–specific qualitative feedback. We hypothesize that more explicit consideration of older adult–sensitive criteria using the Older Adult Fitness Technology Translation Assessment tool, technologies can reduce caregiver burden, lower health care costs by delaying functional decline, and enhance quality of life. Future work is necessary for informing best practices for both the design and assessment of fitness technologies for older adults. An investigation into the role of co-design in aligning fitness technologies with older adult preferences, the best use of older adult fitness technology preference research in design, and a protocol for building toward technology readiness is planned.

A straightforward takeaway from this review is that more explicit inclusion of older adult–specific factors in design and assessment is worth testing to evaluate how these established factors’ integration can support the development of more inclusive and impactful older adult fitness technologies. By integrating the older adult–specific factors summarized in the Older Adult Fitness Technology Translation Assessment into design and assessment methodologies, we hypothesize that we will be much better able to assess the efficacy of the interventions in practice. Furthermore, we hypothesize that it can help bridge the gap between technological capability and real-world applicability, ultimately fostering greater adoption, respect for participants, and long-term success in supporting older adults’ health and well-being.

## Appendix

Multimedia Appendix 1 Summary table of the 43 research papers included in the review. Information included in the table includes the journal publication, system summary, health goal, research goal, study target, number of participants, and intervention duration.

Multimedia Appendix 2 PRISMA-ScR checklist.

Multimedia Appendix 3 The search terms and strategy used for the scoping review process.

Multimedia Appendix 4 The detailed rubric used by reviewers to assess the forty-three papers included in this review across the following eight factors: (1) compatibility with lifestyle, (2) similarity with past experience, (3) dignity and independence, (4) privacy concerns, (5) short term outcomes, (6) long term outcomes, (7) social support, and (8) emotion.

Multimedia Appendix 5 A summary of the cited older adult design works in the 43 reviewed papers and whether the outcomes from those cited works translated to the proposed technology interventions.

Multimedia Appendix 6 Notes from the interrater reliability consensus procedure to resolve disagreements.

## Abbreviations

HCI: human-computer interaction
PRISMA-ScR: Preferred Reporting Items for Systematic reviews and Meta-Analyses extension for Scoping Reviews
TAM: Technology Acceptance Model
UTAUT2: unified theory of acceptance and use of technology extension
VR: virtual reality
