# Supplementary material for: How Well Do Older Adult Fitness Technologies Match User Needs and Preferences? Scoping Review of 2014-2024 Literature
Source: J Med Internet Res. 2025 Sep 24;27:e75667. doi: 10.2196/75667 (PMC12508674; doi:10.2196/75667)
Supplement: Multimedia Appendix 1 [file jmir_v27i1e75667_app1.pdf]

Article	Journal	System Summary	Health Goal	Research Goal	Study Target	# Prts.	Time
Vaziri et al. [48]	IJHC	MS Kinect Exergames	Balance training, fall prevention	Feasibility	65+, living in community	136 (C = 71, I = 63)	16 weeks
	**Outcomes**	Analysis of system effect on subgroup: 1. Age (no difference), 2. Gender (female participants reduced fall risk at higher rate than male counterparts), and 3. IT literacy (main effect of literacy on fall risk reduction)) fall risk. Reduction of fall risk between all groups in comparison to control					
Volders et al. [49]	IERPH	eHealth system w/ wearables	Physical Activity Promotion	Longitudinal study of effects	65+, Dutch speaker, with condition affecting mobility	438 (C = 264, I = 174)	12 months
	**Outcomes**	No effects on objectively measured physical activity, 19.1% and 25.1% dropout at 6 and 12 months respectively, Intervention increased likelihood to perform self-reported cycling, gardening, and walking.					
Van Dyck et al. [50]	JMIR	eHealth w/ wearables (acceleromet er)	Physical Activity Promotion	Longitudinal study of effects	Aged 65 -80	65 (C = 31, I = 68)	5 weeks plus 3 month follow-up
	**Outcomes**	Significant intervention effects on moderate-to-vigorous physical activity (MVPA)					
X. Li et al. [51]	ISMAR	Multisensory VR games	Maintaining visual function, cognitive ability, and physical function in older adults	Acceptability Study	½ Older/ ½ younger adults	28	40 minute acceptabil ity sessions
	**Outcomes**	Suggests that VR game could be well accepted by older adults					
Uzor and Baillie [52]	TOCHI	eHealth system w/ wearables (2 IMU sensors - 9 DOFs)	Rehabilitation and fall prevention	Feasibility	65+, living in own home, fallen in last 12 months, can do home exercise using standard care booklet	38 (C = 22, I = 16)	8-weeks
	**Outcomes**	Significant greater adherence vs. control group, more participants believed they achieved their rehabilitation goals vs. control group, Higher reduction in stride length variability vs. control group (measure of fall risk)					
Nawaz et al. [53]	Pervasive Health	MS Kinect Exergames	Balance training, fall prevention, strength building	Usability	Not provided	12	1 hour sessions

	**Outcomes**	Participant Feedback: Liked games that were related to real life (skiing and tennis) and physically challenging, wanted clear objectives, sense of progress, appropriate music, and a single activity at a time					
Lin et al. [54]	Gerontology	Exergame, ergometer, smart fitness equipment	Physical Activity Promotion	Short Term Randomized Control Trial	50+, active in community	35	12 weeks
	**Outcomes**	Significant improvements in muscle strength, flexibility, and aerobic endurance in comparison to control group					
Petersen et al. [55]	JMU	mHealth (App), smart fitness equipment (exercise band)	Strength building and physical activity promotion	Usability	65+, community dwelling	16	Not reported
	**Outcomes**	Overall System Usability Scale (SUS) and Ease of Use (USE) scores were 66.4 and 41.3 respectively					
Matz-Costa et al. [56]	JGSW	Wearables (FitBit Zip pedometer), mHealth (Tablet app), phone	Physical Activity Promotion, Cognitive activity, and social interaction	Feasibility	65+, relatively inactive, city resident	25 (C = 13, I = 12)	8 weeks
	**Outcomes**	83% retention rate, daily steps increased by 431 steps from baseline to week 4 and sustained at Week 8					
Smaerup et al. [57]	AT	eHealth system	Vestibular rehabilitation, physical activity promotion	Feasibility/ Long-term assessment of behaviour change	Diagnosed with peripheral, central and/or mixed vestibular dysfunction	7	16 weeks
	**Outcomes**	Participants were basically self-efficient and accepted the technology, participants asked for greater variation in the exercises and closer contact with physiotherapist.					
Ogonowski et al. [58]	TOCHI	MS Kinect, Exergame, Senior Mobility Monitor	Balance training and fall prevention	Feasibility/ Long-term assessment	65+	12	6 months
	**Outcomes**	Participants reported a greater awareness of their health and fall risk issues, and reported a feeling of lower fall risk, inactive and active older adults had the largest reduction in fall risk (PPA), 6 month adherence of the system was good, but not all maintained the recommended training schedule of 120 minutes per week					
Skjæret et al. [59]	Gerontology	Exergames, press and release step pad (DDR),	Physical activity promotion	Usability/ design guidelines	65+	14	Not reported

		virtual reality, MS Kinect					
	**Outcomes**	Playing The Mole on SilverFit (virtual reality based game) resulted in the best movement quality with the highest sum score, weight shift, speed variation, and variation in movement direction					
Santini et al. [60]	TBM	mHealth (App)	Wellness motivation, fall prevention, and physical activity promotion	Usability	60+, rural and urban communities	9 (4 full part.)	7 days max
	**Outcomes**	Participants assessed the app as valid, usable, acceptable, and able to sense most reported free-living activities					
McMahon et al. [61]	IERPH	mHealth (App)	Physical activity promotion, mental well-being, and social interaction, transitioning to retirement	Longitudinal study of effects	55+, 3 years pre or post retirement, no care dependency, in good health status condition	62	10-weeks
	**Outcomes**	The use of the digital coach improved participants' physical activity, mental well-being and self-efficacy during the first 5 weeks and only physical activity in the second 5 weeks					
Sit et al. [62]	Frontiers in Public Health	Augmented reality, motion sensors, tablet app (iPad)	Well-being promotion, physical activity promotion	Usability	Not provided	1915	2 minutes
	**Outcomes**	Reported overall satisfaction was high, participants showed enthusiasm using the games					
Muñoz et al. [63]	Sensors	MS Kinect	Physical activity promotion	Usability/ feasibility	Not provided	57	15 days
	**Outcomes**	Progressive acceptance of the system, improvement in measurement of physical achievements					
Knight et al. [64]	The Physician and Sports medicine	mHealth (App), blood pressure monitor, glucometer, and pedometer	Physical activity promotion	Longitudinal study of effects	generally healthy, no diagnosis required	45	12 week
	**Outcomes**	Significant changes from baseline for physical activity, body weight, and blood pressure					

Baranyi et al. [65]	IEEE SeGAH	mHealth (App), serious game	Stroke Rehabilitation	Usability	Not provided	13	Not reported
	**Outcomes**	Some initial difficulties with participant use of game, general positive feedback from participants and therapist involved in study					
M. B. D. Rosario et al. [66]	JBHI	mHealth (App), blood pressure monitor, and weight scale	Cardiac Rehabilitation	Pilot/ feasibility	Referred to CRP for 1st time hospital admission for a cardiac-related diagnosis/ treatment, English speaker	51 (C = 22, I = 29)	6 weeks
	**Outcomes**	88% completion rate in intervention group compared to 67% in control group, statistical correlation found between post six minute walking distance (6MWD) and average time walked each day					
K. Al-Naime et al. [67]	IBIOMED	Wearables, internet of things (IoT)	Preoperative Prehabilitation, rehabilitation	Feasibility	Not provided	17	4-6 weeks
	**Outcomes**	System was able to accurately recognize physical activities at the gym and outdoors, able to facilitate mixed mode prehabilitation program					
S. Mohieldin et al. [68]	IEEE ICC	mHealth (App), Smart fitness equipment (exercise band)	Strength building	Proof of concept/pilot	Not provided	7	Not reported
	**Outcomes**	BandPass had 94% accuracy, recorded 147 exercises, demonstrated ability to facilitate remote monitoring of exercise progress					
S. Kanchanapaetnukul et al. [69]	ITC-CSCC	Video, MS Kinect	Physical Activity Promotion	Preliminary Evaluation, Usability Testing	Elderly (usability testing without elders)	26	Single session
	**Outcomes**	System Results: Precision - 0.94, Recall - 0.98, F1 score - 0.96, Accuracy - 0.93, Ease of use of system statistically better compared to old system					
T. El Salti et al. [70]	IEEE CCWC	mHealth (App)	Physical Activity Promotion	Pilot Usability Study	Actively involved in providing remote and informal care to elderly	12	Single Interview
	**Outcomes**	Participants mentioned that proposed system would strongly support their caregiving of older adults, broad acceptance from participants					

Alizadeh et al. [71]	CHI EA	Wearables (Haptic Feedback)	Physical Activity Promotion	Preliminary Evaluation	Seniors (preliminary testing without seniors)	5	Single session testing
	**Outcomes**	Simple haptic feedback can connect users with remote exercise, vibration useful for sychronization					
Elder et al. [72]	Activities, Adaptation, and Aging	Computer, video	Physical Activity Promotion and Strength Building	Mixed Methods Short Term Pilot Study	50+	10	10 weeks
	**Outcomes**	90% of participants showed improved sit-to-stand (STS) and timed up and go (TUG)					
Ballesteros et al. [73]	Gerontology	Computer, social network, wearables (smart watch), video	Physical Activity Promotion	Longitudinal Controlled Trial	65+	57 (C = 27, I = 28)	12 months
	**Outcomes**	User group showed improvement from pre- test to post test in wellbeing (SPF-IL)					
Coley et al. [74]	JMIR	eHealth	Healthy Ageing, healthy living	Multinational Randomized Controlled Trial	65+, dementia free, community dwellers	2724 (C = 1335, I = 1389)	18 months, 6 month follow up
	**Outcomes**	Compared to control group the adjusted mean difference of blood pressure, BMI, and cholesterol, was −0.08 (−0.12 to −0.03), −0.04 (−0.08 to 0.00), and 0.00 (−0.08 to 0.08) in the high, moderate, and low engagement groups, respectively					
Arkkukangas et al. [75]	PTP	mHealth (App)	Fall Prevention	Qualitative Usability Study	65+, Swedish speaker	12 (7 F, 5 M)	Single day focus groups
	**Outcomes**	Using the application helped all the participants perform the exercises, program was fun to use, monitoring helped participants follow and evaluate their progress regularly and adhere to the exercises					
Gomes et al. [76]	JMIR	Interactive TV App	Healthy Living (Dietary) and Physical Activity Promotion	Feasibility and Acceptability Study	Older adults with food insecurity	31	12 weeks with 3-month follow up
	**Outcomes**	Participants were significantly more interested in using eHealth for food insecurity following intervention, high levels of acceptability were found, reduction of food insecurity, decreased fatigue, and improved physical function					
Hosteng et al. [77]	JPAH	mHealth (App), Wearables (FitBit Zip)	Physical Activity Promotion	Pilot Study	Older Adults living in Retirement Community	54 (42 F, 12 M)	8 weeks, 4 week follow up

			and Social Interaction				
	**Outcomes**	Increased daily steps from 5438/day to 6201/day at week 8, but was not maintained at 4 week follow up following the intervention					
Daly et al. [78]	JMU	Computer, Tablet (App)	Physical Activity Promotion and Strength Building	Short-term Pilot Study	65+, independent living	20	8 weeks
	**Outcomes**	84% adherence, mean weekly walking time increased 78 minutes, MVPA increased 41 minutes, system usability was high					
de Batlle et al. [79]	JMU	mHealth (App), Wearables (FitBit Flex, additional sensors)	Preventative Care for Complex Chronic Patients	Acceptability Study	55+, home dwelling, chronic conditions with history of pulmonary disease or heart failure, or hip/knee surgery	135 (C = 58, I = 77)	3 months
	**Outcomes**	Better continuity of care vs control, high scores for patient acceptability and usability, 77% usage of technology					
Joosen et al. [80]	Frontiers in Digital Health	mHealth (App), Wearables (HR belt)	Physical Activity Promotion	Usability Study	60+, (1/2 self described balance issues)	24 (21 F, 3 M)	Single session focus group
	**Outcomes**	Activity levels increased for first 5 weeks of intervention, slightly decreasing for next 5 weeks (no significant increase in % time active at week 10 vs baseline)					
F. Delmastro et al. [81]	WiMob	mHealth (App), Online/Web/Internet	Long-term care services	Usability Study	Older adults at Nursing home	57 for demo, 10	4 months
	**Outcomes**	Primary Users: consider INTENSA services useful with a average/high level of acceptance to the proposed protocol. Secondary Users (Care Workers): System easy to use					
A. K. Mishra et al. [82]	EMBC	MS Kinect	Remote Physical Therapy (Rehabilitation)	Feasibility/ Validation Study	Not provided	4	Single day session
	**Outcomes**	The therapist rated the technical quality of audio and video and the user satisfaction and PT interaction as high as 4.7 and 4.8 out of 5 respectively					
J. Finkelstein and I. c. Jeong [83]	PHT	Smart exercise equipment (Stationary Bike), HR, BP,	Geriatric rehabilitation	Design Study	Intended for geriatric rehabilitation (volunteers aged 23 to 55 participated)	8	Single day testing

		ECG, web-based					
	**Outcomes**	Technology allows telemedicine interventions supporting safe and efficient exercise at senior patient homes.					
H. G. Zadeh et al. [84]	CISTI	MS Kinect	Physical Activity Promotion, Mental Fitness, Disease Prevention	Feasibility Study	Older adults with Mild Cognitive Impairment (MCI) and Urinary Incontinence (UI)	21	Single day sessions
	**Outcomes**	Participants found the game easy to play, master, and fun to replay the game					
C. L. Petersen et al. [85]	CHASE	mHealth (App), Smart fitness equipment (Resistance Band)	Strength Building	Feasibility Study	65+, who would like to lose weight and improve muscle strength	6 (6 F)	Single day sessions
	**Outcomes**	Can be useful tool that will allow measurement of the number of repetitions per exercise					
J. Doyle et al. [86]	Pervasive Health	Computer (Flash App), wearables (Shimmer kinematic sensors, tracking markers)	Fall Prevention and Balance Training	Usability Study	Older adults	12	2 single day sessions
	**Outcomes**	Participants felt confident could use system in their homes. Import themes: high fidelity animation for visual feedback, integration of multimodal feedback throughout the program, navigation prompts to facilitate moving through the program, allowing participant to control the pace and the importance of progression reports.					
C. L. Petersen et al. [87]	CHASE	mHealth (App), Smart fitness equipment (Resistance Band)	Strength Building	Field-Based Feasibility Study	65+	7 (3 M, 4 F)	1 month (once a week exercises)
	**Outcomes**	Patients were positive about use: patient activation measure: 80.7±14; system usability scale: 6.9±2.9; and confidence in use: 7.7±2.7					
H. B. Jimison et al. [88]	EMBC	MS Kinect, teleconferencing	Physical Activity Promotion	Usability, Engagement, and Adherence Study	Independent Living Elderly Enrolled in Health-	6 (2 M, 4 F)	2-3 weeks

					coaching Program		
	**Outcomes**	Able to maintain adherence to the exercise goals for most participants, Lessons learned: need to switch from computer monitor to digital TVs, difficulty finding room in participants homes, and challenges in using multiple remote control devices					
F. Ofli et al. [89]	JBHI	MS Kinect	Fall Prevention, Strength Building, Physical Activity Promotion	Short-term Field Study	Independent Living Elderly Enrolled in Health-coaching Program	6 (2 M, 4 F)	~ 20 days (10 sessions every other day)
	**Outcomes**	The system able to collect continuous exercise data for all sessions, participants expressed positive attitude toward using such a system					
Uzor and Baillie [90]	IMWUT	MS Kinect	Fall Rehabilitation	2-Part Qualitative Study on Design	65+, fallen at least once in prior 12 months	11 (6 F, 5 M), 16 (10 F, 6 M)	Workshop, 2 month study
	**Outcomes**	High levels of acceptance, usefulness, and usability scores from 2 month user study					
