# Supplementary material for: How Well Do Older Adult Fitness Technologies Match User Needs and Preferences? Scoping Review of 2014-2024 Literature
Source: J Med Internet Res. 2025 Sep 24;27:e75667. doi: 10.2196/75667 (PMC12508674; doi:10.2196/75667)
Supplement: Multimedia Appendix 3 [file jmir_v27i1e75667_app3.pdf]

Initial Search Results and Methods		
IEEE Xplore	("older adults" OR elderly OR geriatric OR senior OR "older people") AND ("Physical Activity" OR exercise OR "training program" OR strength OR fitness) AND (Technology OR "digital health" OR "interactive system")	
Filters	journals	345
ACM Digital Library	("older adults" OR elderly OR geriatric OR geriatrics OR aging OR senior OR seniors OR "older people" OR "aged 65" OR 65+ OR 60+ OR "aged 60") AND ("Physical Activity" OR exercise OR "training program" OR strength OR fitness) AND (Technology OR "digital health" OR "interactive system" OR eHealth OR mHealth)	
Filters	Journal, Older adults section and exercise section much be included in the abstract.	1008
PsycInfo	("older adults" OR elderly OR geriatric OR geriatrics OR aging OR senior OR seniors OR "older people" OR "aged 65" OR 65+ OR 60+ OR "aged 60") AND ("Physical Activity" OR exercise OR "training program" OR strength OR fitness) AND (Technology OR "digital health" OR "interactive system" OR eHealth OR mHealth)	
Filters	Journal Publications	1529
Medline	("older adults" OR elderly OR geriatric OR geriatrics OR aging OR senior OR seniors OR "older people" OR "aged 65" OR 65+ OR 60+ OR "aged 60") AND ("Physical Activity" OR exercise OR "training program" OR strength OR fitness) AND (Technology OR "digital health" OR "interactive system" OR eHealth OR mHealth)	
Filters	Peer-reviewed, Aged 65+, English language	3152
TOTAL	6034	

ROUND 2 Search Results and Methodology		
MEDLINE	AB (("older adults" OR elderly OR senior)) AND AB (("Physical Activity" OR exercise OR "training program" OR strength OR fitness OR "independent living")) AND AB	90 results

	((“digital health” OR “interactive system” OR eHealth OR mHealth))	
filters	Peer Reviewed; Publication Date: 20140101-; English Language; Age Related: Aged: 65+ years	
PSYCINFO	AB ((“older adults” OR elderly OR senior)) AND AB ((“Physical Activity” OR exercise OR “training program” OR strength OR fitness OR "independent living")) AND AB (("technology" OR “digital health” OR “interactive system” OR eHealth OR mHealth))	110 results
filters	Publication Date: 20140101-; Peer Reviewed; English language; Age Groups: Aged (65 yrs & older)	
IEEE Xplore	("All Metadata":"older adults" OR "All Metadata":elder) AND ("All Metadata":"physical activity" OR "All Metadata":exercise OR "All Metadata":"training program" OR "All Metadata":strength OR "All Metadata":fitness OR "All Metadata":"independent living") AND ("All Metadata":"digital health" OR "All Metadata":"interactive system" OR "All Metadata":ehealth OR "All Metadata":mhealth)	40
ACM Digital Library	[[Abstract: ab] OR [Abstract: "older adults"] OR [Abstract: elderly] OR [Abstract: senior]] AND [[Abstract: ab] OR [Abstract: "physical activity"] OR [Abstract: exercise] OR [Abstract: "training program"] OR [Abstract: strength] OR [Abstract: fitness] OR [Abstract: "independent living"]] AND [[Abstract: ab] OR [Abstract: "digital health"] OR [Abstract: "interactive system"] OR [Abstract: ehealth] OR [Abstract: mhealth]]	61

״	[[All: "older adults"] OR [All: elderly]] AND [[All: "physical activity"] OR [All: "independent living"]] AND [[All: "digital health"] OR [All: "interactive system"]] AND NOT [[All: young] OR [All: children]]	127
