# Supplementary figures and images for: How Well Do Older Adult Fitness Technologies Match User Needs and Preferences? Scoping Review of 2014-2024 Literature
Source: J Med Internet Res. 2025 Sep 24;27:e75667. doi: 10.2196/75667 (PMC12508674; doi:10.2196/75667)

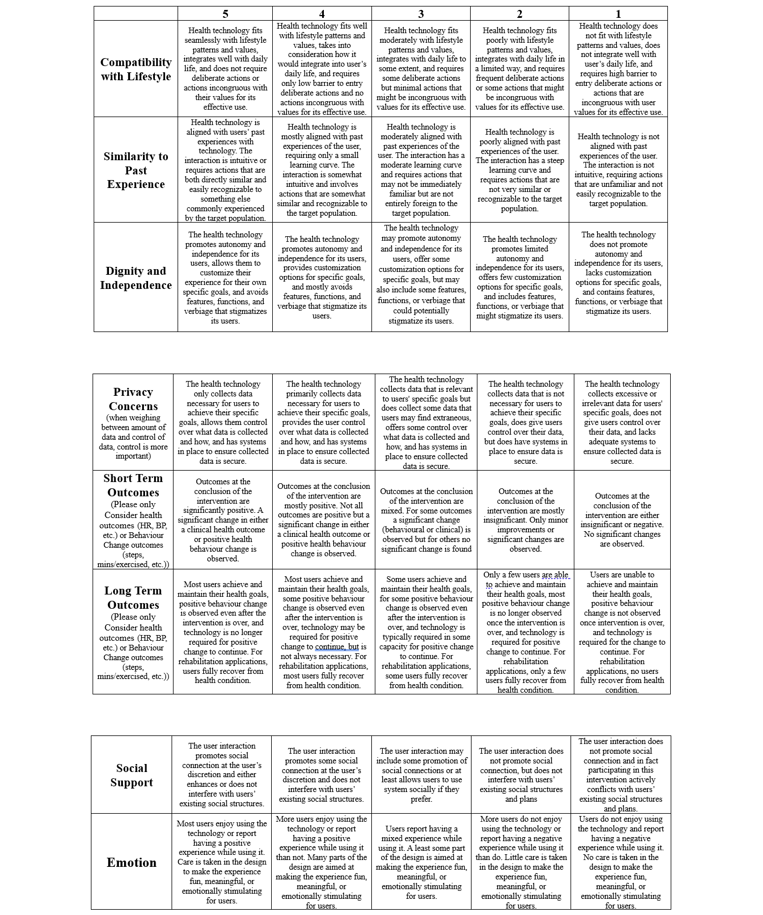

Supplement: Multimedia Appendix 4 [file jmir_v27i1e75667_app4.png]
