# Supplementary material for: How Well Do Older Adult Fitness Technologies Match User Needs and Preferences? Scoping Review of 2014-2024 Literature
Source: J Med Internet Res. 2025 Sep 24;27:e75667. doi: 10.2196/75667 (PMC12508674; doi:10.2196/75667)
Supplement: Multimedia Appendix 5 [file jmir_v27i1e75667_app5.pdf]

Article	Elder Design Works Cited	Where Work was Cited	Major Design Takeaways from Cited Works	Translation of Cited Works to the Design
Vaziri et al. [48]	Sherrington et al. [91]	Design	Fall prevention exercises should have moderate or high challenge, be at least 2 hours per week, both group or home-based programs should be available, could incorporate strength training	Somewhat (180 minutes per week, at home exercise, strength training included, no option of group based training)
Volders et al. [92]	Boekhout et al. [93]	Background, Design, Methods	Include both at home and outdoor activities, stress long term health, stress social interaction, encourage advice from GP and physiotherapist, inform of exercise possibilities, give chose of delivery method between web and printed materials	Yes (Directly developed ActivePlus 65 system from these requirements)
Van Dyck et al. [50]	Did not Cite Elder Design Works	N/A	N/A	N/A
X. Li et al. [51]	Did not Cite Elder Design Works	N/A	N/A	N/A
Uzor and Baillie [52]	1. Alankus et al. [94]<br>2. Uzor et al. [95]	Background, Design	1. Assume no use of hands, simple games, less repetitive and more enjoyable exergames, 2. convey progress,	Yes (Exergames directly designed through the cited works)

Nawaz et al. [53]	Gerling et al. [96]	Design	Exergames should be able to be played both standing up and sitting down, avoid too extensive and sudden movements, have adjustable difficulty level, simple interactions, constructive feedback	Mostly (simple, "limited information" exergames, constructive feedback, not enough information on the required interactions)
Lin et al. [54]	Did not Cite Elder Design Works	N/A	N/A	N/A
Petersen et al. [55]	Batsis et al. [97]	Design	Barriers include privacy concerns, access to tech, and complexity, Enablers: motivation, tracking, accountability	Yes (simple, includes progress tracking)
Matz-Costa et al. [56]	Did not Cite Elder Design Works	N/A	N/A	N/A
Smaerup et al. [57]	1. Phillips et al. [98]<br>2. Hensel et al. [99]	Background, Design	1. Adapt activity programs to enable less able-bodied people, facilitate empowerment, focus on accessibility and affordability, promote socialization, 2. Health technologies should minimize obtrusiveness to end users (physical, usability, privacy, function, interaction, self-concept, routine, and sustainability)	Somewhat (adaptable exercises, some issues with technology uptake, functionality, and motivation, also potential privacy concerns)

Ogonowski et al. [58]	Meurer and Wieching [100]	Background, Design, Methods	1. Design should focus on the necessary feature for training over nice to have community features, eldders preferred personal contact over virtual social interactions, 2.	[No] (Complex technology design with many components, virtual social media platform)
Skjæret et al. [59]	Did not Cite Elder Design Works	N/A	N/A	N/A
Santini et al. [60]	McLaughlin and Pak [101]	Design, Methods	Usability requirements for displays and user interfaces for older adult users	Yes (Directly informed design and approach)
McMahon et al. [61]	Did not Cite Elder Design Works	N/A	N/A	N/A
Sit et al. [62]	Did not Cite Elder Design Works	N/A	N/A	N/A
Muñoz et al. [63]	De Angeli et al. [102]	Background	1. Avoid protrays elders as in decline, ageing technologies should promote resilience, belonging, autonomy, feeling good, and doing good. 2.	Unknown (Not enough information on the promotion of resilience, belonging, and autonomy)
Knight et al. [103]	Did not Cite Elder Design Works	N/A	N/A	N/A
Baranyi et al. [65]	Did not Cite Elder Design Works	N/A	N/A	N/A
M. B. D. Rosario et al. [66]	Did not Cite Elder Design Works	N/A	N/A	N/A
K. Al-Naime et al. [67]	Did not Cite Elder Design Works	N/A	N/A	N/A

S. Mohieldin et al. [68]	Did not Cite Elder Design Works	N/A	N/A	N/A
S. Kanchanapaetnukul et al. [69]	Did not Cite Elder Design Works	N/A	N/A	N/A
T. El Salti et al. [70]	Did not Cite Elder Design Works	N/A	N/A	N/A
Alizadeh et al. [71]	Did not Cite Elder Design Works	N/A	N/A	N/A
Elder et al. [72]	Did not Cite Elder Design Works	N/A	N/A	N/A
Ballesteros et al. [73]	Did not Cite Elder Design Works	N/A	N/A	N/A
Coley et al. [74]	Akenine et al. [104]	Design, Methods	Engagement in self management prevention programmes is influenced by access to reliable information, trust in healthcare providers, and burden of dementia and stigma --> eHealth interventions should be reliable, accessible, and safe	Yes (Information presented was reliable and accessible)
Arkkukangas et al. [75]	Did not Cite Elder Design Works	N/A	N/A	N/A

Gomes et al. [76]	Did not Cite Elder Design Works	N/A	N/A	N/A
Hosteng et al. [77]	Chen [105]	Background	Barriers to physical activity include physical health problems, fear, past sedentary lifestyle, and lack of understanding, effective interventions should reduce these barriers and provide a supportive environment	Unknown (Not enough information on how the intervention reduced the outlined barriers)
Daly et al. [78]	1. Ziebart et al. [106]<br>2. Yardley et al. [107]	Background	1. Barriers to physical activity include lack of knowledge on exercises, fear of fracturing, and trust in providers. Interventions should be tailored to gender, age, or presence of comorbid conditions. 2. Elders preferred to carry out strength and balance training at home	Yes (At home strength and balance training with exercise program)
de Batlle et al. [79]	Did not Cite Elder Design Works	NA	N/A	N/A
Joosen et al. [80]	Kim et al. [108]	Background	Difficulties encountered by elders using IT: physical and cognitive changes, difficulty learning new tasks on mobile phones	Unknown (Not enough information to determine whether the technology addresses the elder specific difficulties cited)
F. Delmastro et al. [81]	Did not Cite Elder Design Works	N/A	N/A	N/A
A. K. Mishra et al. [82]	Did not Cite Elder Design Works	N/A	N/A	N/A

J. Finkelstein and I. c. Jeong [83]	Did not Cite Elder Design Works	N/A	N/A	N/A
H. G. Zadeh et al. [84]	1. McLaughlin and Pak [101]<br>2. Fisk et al. [109]	Background	1. Display and user interface guidelines for elders, 2. Vision, hearing, and haptics affect user interaction, design of interfaces, 3.	Unknown (Not enough information)
C. L. Petersen et al. [85]	Did not Cite Elder Design Works	N/A	N/A	N/A
J. Doyle et al. [86]	Did not Cite Elder Design Works	N/A	N/A	N/A
C. L. Petersen et al. [87]	Did not Cite Elder Design Works	N/A	N/A	N/A
H. B. Jimison et al. [88]	Did not Cite Elder Design Works	N/A	N/A	N/A
F. Ofli et al. [89]	Ijsselsteijn et al. [110]	Background, Design	1. Interface design for elders should minimize burden on spatial memory, working memory, visual functions, and motor ability. Interfaces should be adaptable. 2. Therapeutic exergames should be support a wide range of exercises, hands free,	Somewhat (Supports range of exercises, but includes fixed in exercise feedback)

			minimal number of sensors, adaptable feedback, customizable	
Uzor and Baillie [90]	1. Alankus et al. [94]<br>2. Bongers et al. [111]	Background	1. Assume no use of hands, simple games, less repetitive and more enjoyable exergames, 2. Three main issues affecting rehabilitation: motivation, customisation, independence	Unknown (Not enough information on the user experience)
