# Supplementary material for: How Well Do Older Adult Fitness Technologies Match User Needs and Preferences? Scoping Review of 2014-2024 Literature
Source: J Med Internet Res. 2025 Sep 24;27:e75667. doi: 10.2196/75667 (PMC12508674; doi:10.2196/75667)
Supplement: Multimedia Appendix 6 [file jmir_v27i1e75667_app6.pdf]

Consensus Ratings for Flagged Interrater Disagreements

Current Weighted Kappa Scores (% agreement removing agreement due to random chance with quadratic weighting for larger disagreements)

Compatibility with Lifestyle – 0.448 (Moderate)

Similarity with Past Experience – 0.301 (Fair)

Dignity and Independence – 0.145 (Slight)

Privacy – 0.204 (Slight)

Short-term Outcomes – N/A

Long-term Outcomes – N/A

Social – 0.299 (Fair)

Emotion – 0.421 (Moderate)

Papers with Disparate Scores

1. Implementing Mobile Health-Enabled Integrated Care for Complex Chronic Patients: Patients and Professionals' Acceptability Study.
    a. Similarity with Past Experience – R1 (5), R2 (2)
        i. Notes: Asks to interact with many components, some sensors wouldn't be familiar with daily use but would have experience using with a health provider.
        ii. New Score: 3
2. Evaluation of an mHealth-Based Adjunct to Outpatient Cardiac Rehabilitation
    a. Privacy – R3 (1), R4 (4)
    b. Notes: NFC (Near field communications) more protected and voluntary, collected a lot of data
    c. New Score: 3
3. Haptics in remote collaborative exercise systems for seniors
    a. Privacy – R2 (N/A), R1 (4)

b. Short-term Outcomes – R2 (3), R1 (N/A)
c. Notes: No results, only collecting arm movement data
d. New Score: Short-term outcomes (N/A), Privacy (4)

4. Development and preliminary validation of an interactive remote physical therapy system
    a. Privacy – R2 (1), R1 (5)
    b. New Score (3) - Only collect position data but mention using public server
5. Analysis of effects and usage indicators for an ICT-based fall prevention system in community dwelling older adults
    a. Short-term Outcomes – R3 (4), R2 (N/A)
    b. New score (4) - Significant reduction in fall risk
6. Feasibility, Usability, and Enjoyment of a Home-Based Exercise Program Delivered via an Exercise App for Musculoskeletal Health in Community-Dwelling Older Adults: Short-term Prospective Pilot Study.
    a. Short-term Outcomes – R2 (N/A), R1 (4)
    b. New score (3), increases in physical activity, walking time, but physical function not significant
7. Mr Dustman a Multitasking Digital Game for Older Adults who Suffer from Mild Cognitive Impairment and Urinary Incontinence
    a. Short-term Outcomes – R2 (5), R4 (N/A)
    b. New Score (N/A) - Only design feedback
8. Detection and Monitoring of Repetitions Using an mHealth-Enabled Resistance Band
    a. Short-term Outcomes – R2 (4), R1 (N/A)
    b. Short term Outcomes (N/A), only feasibility results
    c. Emotion – R2 (4), R1 (1)

   d. New score (1), no design elements aimed at emotion

9. Remote Rehabilitation: A Field-Based Feasibility Study of an mHealth Resistance Exercise Band Demo
   a. Short-term Outcomes – R2 (4), R1 (N/A)
   b. Short term Outcomes (N/A)
   c. Emotion – R2 (4), R1 (1)
   d. Emotion (1) ---> same as previous
10. Remote health coaching for interactive exercise with older adults in a home environment
    a. Short-term Outcomes – R2 (4), R1 (N/A)
    b. New score (N/A) - only usability and feasibility
11. Factors Predicting Engagement of Older Adults With a Coach-Supported eHealth Intervention Promoting Lifestyle Change and Associations Between Engagement and Changes in Cardiovascular and Dementia Risk: Secondary Analysis of an 18-Month Multinational Randomized Controlled Trial.
    a. Long-term Outcomes – R2 (3), R1 (N/A)
    b. New Score (3), reduction in engagement in long term but those engaged found improvement
    c. Emotion – R2 (1), R1 (N/A)
    d. New score (3), design decisions aimed to motivate participants
12. A Home-Based eHealth Intervention for an Older Adult Population With Food Insecurity: Feasibility and Acceptability Study.
    a. Long-term Outcomes – R2 (4), R1 (N/A)
    b. New score (N/A) - three month study

13. Multilevel mHealth Intervention Increases Physical Activity of Older Adults Living in Retirement Community.
    a. Long-term Outcomes – R2 (1), R4 (1/N/A)
    b. New Score (1) - Physical activity goes back down to baseline
    c. Social – R2 (5), R4 (2)
    d. New score (4)
14. A smartphone-based solution to monitor daily physical activity in a care home.
    a. Long-term Outcomes – R2 (N/A/2), R4 (N/A/1)
    b. New score (N/A) - 10 week intervention
15. Exploring the Communication of Progress in Home-based Falls Rehabilitation using Exergame Technologies
    a. Long-term Outcomes – R2 (NA/2), R5 (N/A)
    b. New score (N/A) - 2 month study, no outcome metrics collected
16. An ICT-mediated social network in support of successful ageing
    a. Emotion – R2 (3), R1 (N/A)
    b. New score (3), some elements aimed at motivating users in UI
17. BASE - An interactive technology solution to deliver balance and strength exercises to older adults
    a. Emotion – R2 (5), R4 (1)
    b. New score (4) - Added real-time feedback and tracking elements
